# Classification of static postures with wearable sensors mounted on loose clothing

**DOI:** 10.1038/s41598-022-27306-4

**Published:** 2023-01-04

**Authors:** Udeni Jayasinghe, Balazs Janko, Faustina Hwang, William S. Harwin

**Affiliations:** 1 Biomedical Engineering Section, University of Reading, Reading, RG6 6DH UK; 2 University of Colombo School of Computing, Information Systems Engineering, Colombo, Sri Lanka; 3 RACE, UKAEA, Culham Science Centre, Abingdon, OX14 3DB UK

**Keywords:** Biomedical engineering, Computer science

## Abstract

Inertial Measurement Units (IMUs) are a potential way to monitor the mobility of people outside clinical or laboratory settings at an acceptable cost. To increase accuracy, multiple IMUs can be used. By embedding multiple sensors into everyday clothing, it is possible to simplify having to put on individual sensors, ensuring sensors are correctly located and oriented. This research demonstrates how clothing-mounted IMU readings can be used to identify 4 common postures: standing, sitting, lying down and sitting on the floor. Data were collected from 5 healthy adults, with each providing 1–4 days of data with approximately 5 h each day. Each day, participants performed a fixed set of activities that were video-recorded to provide a ground truth. This is an analysis of accelerometry data from 3 sensors incorporated into right trouser-leg at the waist, thigh and ankle. Data were classified as static/ dynamic activities using a K-nearest neighbour (KNN) algorithm. For static activities, the inclination angles of the three sensors were estimated and used to train a second KNN classifier. For this highly-selected dataset (60000–70000 data points/posture), the static postures were classified with 100% accuracy, illustrating the potential for clothing-mounted sensors to be used in posture classification.

## Introduction

Maintaining correct posture in daily life is important and brings benefits such as maintaining good blood circulation and reducing the risk of chronic diseases^1,2^. When it comes to healthcare monitoring systems, for example in rehabilitation settings, it is important to monitor posture as well as the daily activity intensity of an individual^3,4^. Such monitoring allows both the person and the healthcare professional to assess the condition and the effects of any interventions, thereby helping to avoid injuries such as those arising from falls and to improve the physical condition of the patient^5^. For example, in stroke rehabilitation, posture evaluation can be done in the clinic using the Postural Assessment Scale for Stroke Patients (PASS)^6^ and can be used to measure the progress of patients’ recovery^7^. The availability of a PASS-like measurement with a finer graticule and greater accuracy can provide better insight into this recovery.

Mosenia et al.^8^ noted posture identification and posture correction as some of the main applications of wearable medical sensors. Commercially-available wearable sensors are popular in activity monitoring in free-living environments as they can be used as self-monitoring devices. Consumer products typically contain all their sensors in a single housing designed to be worn in one body location, for example, on the wrist. However, research into activity-^4,9,10^ and posture-classification^11–13^ has demonstrated that the use of multiple sensors increases classification accuracy. Further, there is a trade-off between having multiple sensors with light-weight algorithms and having a single sensor to extract multiple heuristic features to feed into a complex algorithm.

For the end user, putting on multiple sensors can be a tedious or laborious task, and this can be exacerbated when the physical process of attaching the sensor to the body is difficult, for example due to motor impairment or due to a design requiring good manual dexterity. Furthermore, analysis of the sensor data can be complicated if sensors are incorrectly placed, or if they slip off the limb during the day. One approach to make it easier for the end user to wear multiple sensors is to embed the sensors into garments^13–15^. In 2002, Laerhoven et al.^16^ emphasised the importance of mounting sensors into clothing. They claimed that as clothing gives a larger space to mount multiple miniaturised IMUs, clothing is an excellent platform to collect more data without disturbing the wearers. Most prior work on smart garments investigates tight-fitting garments in order to hold the sensors in-place close to the body. Our research investigates sensors in loose-fitting, everyday clothing that is likely to be more comfortable for the wearer, easier to don on and off and more appropriate for everyday use.

This work is novel in a number of ways. Most prior work in smart garments investigates tight-fitting garments, whereas here we investigate loose clothing which is likely to be more comfortable for everyday use. Other studies investigating loose clothing have examined specific activities (shoulder movements^17^) or specific clothing items (hospital garments^18^), whereas here the sensors are embedded into participants’ own everyday clothing and capture data relating to a range of everyday activities/postures. Our study was conducted in a semi-natural setting over an extended period (hours and days), compared with others^15,17–19^ which look at shorter time periods (e.g. on the order of minutes) under controlled conditions. Finally, this study demonstrates that a single feature from each of multiple sensors is enough to achieve a high level of classification accuracy.

## Related work

Work by Lyons et al.^12^ describes a method of calculating the inclination angles of the thigh and trunk for different postures. Dual axis accelerometers were strapped onto the person over their clothing. Data analysis distinguished activities into dynamic and static activities based on the standard deviation of the magnitude of the thigh accelerometer data over a 1 second window. Lyons et al. used a pre-defined threshold value to assign the data into the two categories (static and dynamic)^12^. Vipul et al.^11^ used both waist and thigh sensor data (right hand side) to classify data into dynamic and static activities. They investigated two features, the integral of the signal magnitude over 1 s windows and a continuous wavelet transform of the filtered raw acceleration. Fida et al.^20^ described how the window sizes contribute in identifying static/ dynamic/ transition activities. Their conclusion was that larger window sizes (e.g. 1.5–3.0 s) gave higher accuracy in identifying long duration activities and smaller window sizes (e.g. 0.5 s), gave higher accuracy in identifying short duration activities such as transitions^20^. Fida et al. used a single triaxial accelerometer mounted on the waist and a feature vector consisting of 22 time-based components, including means, standard deviations, skewness, and kurtosis for all window sizes (0.5 s, 1s, 1.5 s and 3 s). Chong et al.^21^ examined 206 time and frequency-based features with different types of classifiers (Artificial Neural Networks, Support Vector Machines and Random Forests(RF)) in activity classification with a single accelerometer mounted on the right hip. The study indicated that subsets from time-domain features are sufficient to classify accelerometry into activities even without analysing frequency domain features^21^.

Other than the above mentioned studies with body-mounted sensors and smart-textile data, Chiuchisan et al.^18^ used a pair of loose-fitting trousers with an Arduino Nano board with 2 inductive sensors and an IMU. The sensor was placed near the knee. From their data, they concluded that there was potential to use these data from loose-fitting trousers for identifying different movement patterns in clinical rehabilitation^18^.

In addition to the studies based on data from the lower-body, data from the upper body has also been investigated in posture classification. Lin et al.^19^ and Harms et al.^17^ implemented posture classifiers with sensors mounted in loose-fitting jackets. The study conducted by Lin et al. was based on four low-cost strain sensors mounted on the shoulder, elbow, abdomen and waist^19^. They used Long Short-Term Memory (LSTM) networks in implementing three classifiers with their sensor output which was a single voltage value. First, they classified the data into three static postures (standing, sitting and lying) and two dynamic activities (walking and running). Secondly, they detected static postures with random arm movements and finally, they classified the data into sitting and two different slouch positions^19^. Harms et al. attached accelerometers onto the forearm and upper arm of a loose-fitting garment to classify the data into ten postures that are useful in shoulder and elbow rehabilitation. They implemented a simulation with a body model and corrected the orientation error based on empirical samples of data. Finally, they concluded that there was a possibility of increasing the classifier accuracy based on the correction of the simulation^17^.

To perform posture classifications, both^11^ and^12^ calculate inclination angles of the waist and thigh sensors relative to the direction of gravity. Skach et al.^15^ use woven pressure sensors on trousers to categorise postures. These sensors were near the person’s thigh and buttocks during a set of video-recorded, controlled postures involving the thigh and shank (e.g. leg-crossing postures). They used a RF classifier to classify the data into postures^12^ and^15^ both relied on thigh data in posture classification.

In^11,12^ the sensors were strapped onto the body over the clothing and in^15^ the sensors were woven into the clothing. In our present study, the sensors were attached to the inside of loose clothing. Prior to the work reported here, an earlier study verified how well loose clothing-mounted sensor data correlated with body-worn sensor data^22^ and concluded that clothing sensor data were reasonably correlated with body worn sensor data, especially with static postures. Hence, there is good potential of using clothing-mounted sensor data in activity/posture classification.

The above mentioned studies which used lower-body data in posture classification^11,20,21^ used multiple features from each sensor to train the classifiers. Even though Lin et al.^19^ used only a single feature from each sensor, they used an LSTM (deep learning approach) network in their study. Rather than using a deep learning approach which usually trains classifiers on the raw data, our study uses a machine learning approach with a meaningful single feature (inclination angle). As the inclination angles of body parts are used as the feature vector, the features can be easily represented by a stick figure for an intuitive interpretation of the classifier output. Vipul et al.^11^ (waist and thigh) and Lyons et al.^12^ (trunk and thigh) used two body-mounted sensors in their studies. However, an additional ankle sensor, as studied here, can improve classification accuracy as it helps to differentiate postures that depend on the lower leg. Further, Lyons et al. pre-defined threshold values for the inclination angles to classify the data into different postures, whereas the present study uses a machine-learning approach to define the classes, rather than hard coding the threshold values. See supplemental files for a table comparing prior studies with the present work.

## Methodology

### Materials

**Figure 1:**
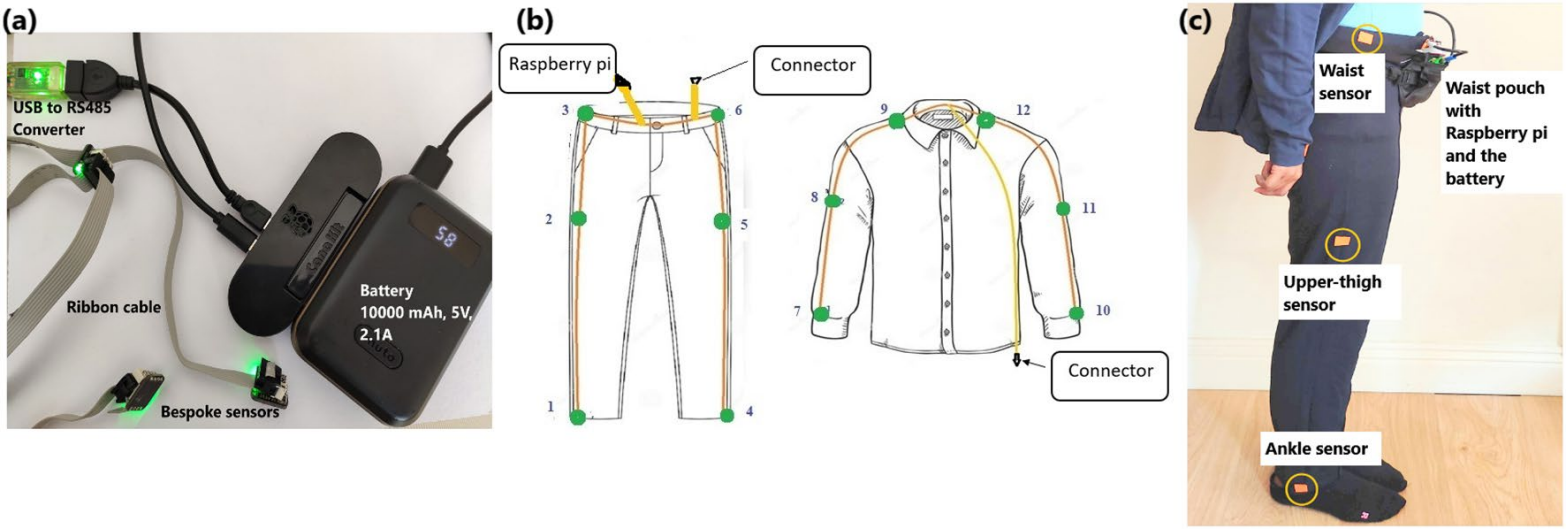
(**a**) Components of the sensor system. (**b**) Sensor placement on clothes. (**c**) Sensor placement on trousers. 12 IMUs are connected to a synchronous bus via ribbon cable. The sensors are connected to a Raspberry Pi via a USB to RS485 converter, and the Pi was powered by a battery pack. Lights on the sensors provided assurance of the sensors’ operation but were not visible outside the clothing. The Pi and battery were worn in a waist-pouch attached with a belt, with 4-pin connectors connecting the bag components, the trousers and the top. The 12 sensors were sampled synchronously at 50Hz. Since accelerometer, magnetometer, and gyroscope data were all recorded from each sensor, this demanded a time division multiplexing bus protocol running at 500K baud.

Our sensing system consisted of 12 IMUs (based on the Bosch Sensortec BMI160 smart IMU), all using a differential serial bus, connected using flat ribbon cable forming a *“sensor string”*. The 12 bespoke sensors were approximately 15$\times$12$\times$7 mm each (see Fig. 1a) and had a combined weight of 18g. The inter-connecting cables weighed 146 g. The sensor string was connected to a Raspberry Pi where the data were stored (Fig. 1a). A battery pack enabled continuous mobile data collection for more than 12 hours (10,000 mAh output : 5V, 2.1A). Data were sampled at 50 Hz. The range of the accelerometers was ± 16 g with 12-bit resolution. The BMI160 IMU includes a gyroscope and a magnetometer, which were also used to record data, alongside the accelerometers. The sensors use a time division multiple access (TDMA) based protocol, where each start-of-frame character allows the IMUs to trigger the acquisition of the next sample, thus resulting in a tightly-synchronised sensor network.

The 12 IMUs were positioned in the clothing so that there were sensors along the lateral side of the upper limbs (wrist, upper arm, and shoulder/neck) and lower body (ankle, thigh, waist), on both sides (Fig. 1b). To attach the sensors to the clothes, the sensors were taped securely along the inner seams of the clothes in the chosen position, and cotton bias binding was taped on top of the sensor string using double-sided tape for fabric. In this way, the sensors were not outwardly visible (see Fig. 1c) and also not in contact with the skin which helped to make the system more comfortable for the wearer.

### Data collection procedure

Five healthy participants (age range: 28–48 years old; 3 males and 2 females) took part in the study. Each person selected a pair of trousers and a hoodie jacket in their usual size, and the researcher attached the sensors to the clothes. Four participants wore cotton-blend fleece jogging trousers, and one wore loose cotton slacks. Participants wore the clothes on three or four days (with the exception of 1 participant who was only able to take part for one day) for 5–8 hours per day of data collection. The Raspberry Pi and the battery pack were kept in a bag on the waist of each participant as shown in Fig. 1c.

On each day of data collection, participants were asked to perform a set of predefined activities which were videoed to provide a ground truth. Thereafter they continued with their usual activities for the rest of the day. The ground truthed activities comprised two minutes of each of the following: (1) Standing still, (2) Sitting on a chair, (3) Lying on their back (supine position), (4) Sitting on the floor with legs outstretched, (5) Walking back and forth and (6) Going up and down stairs. For the rest of the day’s activities, participants were requested to keep a diary of their activities.

The study was reviewed by the research ethics committee of the School of Biological Sciences, University of Reading, UK and given a favourable ethical opinion for conduct (reference: SBS-19-20 31). The study was conducted in accordance with this approved protocol and the relevant guidelines and regulations. All participants provided written informed consent.

### Data processing work flow

In this section, we provide an overview of the data processing workflow. Further elaboration of particular steps in the workflow are in the subsections that follow.

Data from the 12 IMUs were logged onto the Raspberry Pi. Once the data collection was completed, the data were transferred to a PC and analysed using MATLAB. Although the IMUs provide accelerometer, gyroscope and magnetometer readings, the main focus of this analysis was the accelerometer data from the right side of the lower body (sensors 1, 2 and 3 as shown in Fig. 1b).

As the placement and orientation of the sensors relative to the body could vary slightly from day to day depending on the fit of the clothing, each day’s data were pre-processed to align all sensors to a common coordinate frame such that the z-axis is aligned with the direction of gravity  (Section ‘Data pre-processing’). The data were also low-pass filtered to remove noise. From the filtered data, the sensor-to-vertical angle was estimated as described in Section ‘Calculating sensor to vertical axis angles’.

A classifier was implemented to first classify the data into “static postures” or “dynamic movements” (Section ‘Classifying static and dynamic activities (classifier 1)’). For data classified as “static postures”, a second classifier was implemented to classify the data into four specific postures (i.e. standing, sitting on a chair, lying down, sitting with legs outstretched) (Section ‘Classifying postures (classifier 2)’).

### Data pre-processing

#### Data rotations

With the sensors embedded in the clothing, the initial orientation of the sensor relative to the limb and to the world is unknown. Hence, we apply a rotation to the accelerometer data to align the sensor’s z-axis with the direction of gravity. This rotation can be computed easily using Rodrigues’ rotation formula^23^ by identifying the axis for rotation as being perpendicular to both the gravity vector and the z-axis. This is identified as the cross product between the gravity vector and the z-axis and the angle for rotation is the angle between these two vectors. To do this, we find a segment of ‘standing still’ data and assume that the limbs are all vertical and the only accelerations are those due to gravity^22^.

There is a possibility of having a second rotation that transforms the data so that the transformed y-axis is aligned with the anterior-posterior direction and the transformed x-axis is aligned with the medial-lateral direction perpendicular to the sagittal plane. The rotation matrix can be estimated by finding suitable segments of data where there is rotation in the sagittal plane (e.g. walking, leg raising, sitting-to stand). However, the second rotation was not required for the present analysis.

#### Filtering

Accelerometer signals were then low-pass filtered with a second-order Butterworth filter with a 3 Hz cut-off as suggested in^12^. The filter was run on the data both forwards and backwards to minimise phase distortions at the expense of causality.

### Calculating sensor to vertical axis angles

Estimation of the orientation of a wearable inertial sensor from gyroscope, accelerometer and magnetometer measurements is complex, with a variety of approaches^24^. A common simplification is to estimate the sensor inclination angle with respect to the local gravity vector. This estimate can be made with only the accelerometer, but additional information from the gyroscope and magnetometer can be used to improve the estimate of the sensor inclination angle.

The acceleration measured by the accelerometer can be considered as a baseline gravitational acceleration $\textbf{g}$ with a ‘dynamic’ acceleration $\textbf{a}$ added. Thus the accelerometer sensor measurement is $$\begin{bmatrix} s_x,&s_y,&s_z \end{bmatrix}^T = {}^S\textbf{g} + {}^S\textbf{a}$$, where the two accleration components are measured in the sensor frame $\{S\}$^25^. It is fair to assume that the magnitude of gravity is fixed in a world frame $\{W\}$, i.e. $${}^W\textbf{g}\approx \begin{bmatrix}0&0&9.8 m/s^2\end{bmatrix}^T$$ and that for typical human movement the ‘dynamic’ acceleration will have a zero mean if estimated over a sufficiently long time window.

**Figure 2:**
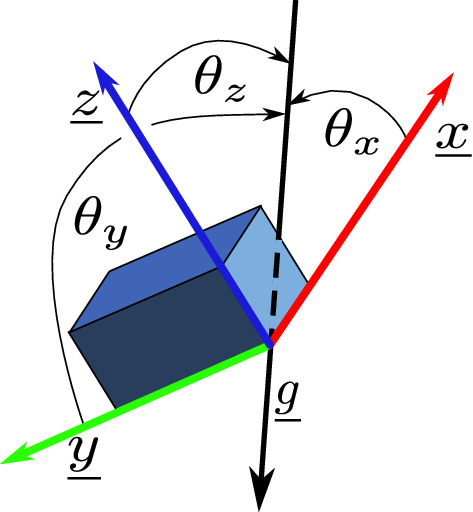
Orientation of the IMU with respect to a vertical axis represented by gravity ($\textbf{g}$) can be defined as the cosines of angles $\theta _x,\theta _y$ and $\theta _z$.

When subjected only to gravitational acceleration (that is, $\textbf{a}=0$) the sensor will measure the components of $\textbf{g}$ in the world frame along its three sensor frame axes as shown in Fig. 2. That is to say that the sensor will measure just the gravity vector so $$\textbf{g}=|\textbf{g}|\begin{bmatrix} \cos {\theta _x}&\cos {\theta _y}&\cos {\theta _z} \end{bmatrix}^T$$.

Three methods are outlined to recover the sensor orientation with respect to gravity and hence the angle of a limb with respect to a vertical axis. The first of these (the $\arccos$ method), was used for subsequent results.

#### Estimating sensor inclination angle with $\arccos$

If the sensor consists of only a 3-axis accelerometer, and we assume one of the sensor axes is aligned with the limb (it was assumed the sensor z-axis aligns with limb), then the inclination of the sensor is simply calculated as the $\arccos$ of the relevant sensor component on the assumption that there is no dynamic (non gravitational) acceleration (Eq. 1).

1 $$\begin{aligned} \theta _z=\arccos \Big (\frac{s_{z}}{|\textbf{g}|}\Big ) \end{aligned}$$

#### Estimating sensor inclination angle with $\textrm{atan2}$

If the sensor is well positioned on the limb so that it is aligned with an anatomical plane (for example the y-axis lies in the sagittal plane with the x-axis perpendicular to the plane), then an $\textrm{atan2}$ function can give information relating to the limb angle (Eq. (2)). Using the $\textrm{atan2}$ function would allow a direct distinction of whether the person was leaning forward or backward, or lying in a supine position or prone position.

2 $$\begin{aligned} \theta _z=\textrm{atan2} \Big ({s_{y}},{s_{z}}\Big ) \end{aligned}$$

This method confines the result to be in the $y-z$ plane that may move with respect to the sagittal plane if the sensor is able to twist on the clothing, hence was not used for this analysis.

#### Estimating sensor inclination angle with a rotation matrix

Sensor fusion algorithms such as MARG (Magnetic, Angular Rate, and Gravity) algorithms attempt to calculate an orientation matrix or quaternion relating the sensor frame $\{S\}$ to the world frame $\{W\}$, for example Mahony et al.^26^, Madgwick et al.^27^ and Sabatini^28^. The orientation of the sensor is then simply the relevant column of the orientation matrix since the gravity vector would align with a world coordinate frame. A problem is that the sensor is defined with respect to a global coordinate frame (e.g. East, North, Up). The ‘horizontal’ (East and North) axis estimates tends to be poor as they suffer from problems such as integration drift or local distortions in the earth magnetic field. It then becomes difficult to align the global frame with the sagittal plane of the individual, hence the $\arccos$ method was considered the simplest and easiest method to use for this work.

### Classifying static and dynamic activities (Classifier 1)

#### Activity extraction

The ground truth videos were synchronised with the sensor data using ELAN software^29^. The start and end points for each of the four static postures (standing, sitting, sitting on the floor with legs outstretched and lying down) and dynamic activities (walking, going up and down stairs) were manually identified and annotated by the first author, and those segments of the data were extracted for analysis. In this way the transitions in-between the activities were deliberately not included in the analysis. For postures and dynamic activities, data segments of approximately 90 s were extracted from longer continuous data segments. The final labelled dataset comprised data collected over 15 participant-days across the 5 participants, with 6 video ground truthed activities per participant per day. Roughly 405,000 $(=6 \text { activities } \times 90 \text { s } \times \text { 50 Hz } \times 15 \text { days})$ data-points were used in the training process (data collection frequency was 50 Hz).

#### Static postures versus dynamic movements

The main intention of this study was to analyse the postures of the participants, hence the activities were first categorised into two classes: static postures and dynamic movements. We extracted three features which were moving standard deviation of the vertical axis of the ‘Thigh’ data and moving standard deviation of the magnitude of the ‘Thigh’ and of the ‘Waist’ data, according to the literature (^11,12,15,21^). Instead of defining threshold values to distinguish static vs dynamic activities as in^11,12^, we compared the accuracy of classifiers in distinguishing the two classes with different combinations of features and with five different window sizes (0.5 s, 1 s, 1.5 s, 2 s and 3 s). The annotation files described in section ‘Classifying static and dynamic activities (classifier 1)’ were used to provide the ground truth for the classifier. The activities were labelled as static (standing still, sitting on a chair, lying on their back, sitting on the floor with legs outstretched) or dynamic (walking back and forth, going up and down stairs). Those labels, along with the three features, were then passed into MATLAB’s ‘Classification Learner App’. The data were trained with all the options available in MATLAB’s ‘Classification Learner App’. These included ‘Discriminant analysis’, ‘Naive Bayes’, ‘Decision trees’, ‘Support vector machines’, ‘K-nearest neighbour (KNN)’ and ‘Ensemble classifiers’. It was found that the ‘Weighted KNN’ classifier achieved the highest accuracy in this classifier (Classifier 1). As such Weighted KNNs which use an Euclidean distance metric with 10 neighbours were used, and a further comparison to study the accuracy of the classifier with different combinations of features and window sizes was conducted. To evaluate the model, 5-fold cross-validation and leave-one-subject-out methods were used.

When analysing the data from the non-ground truthed (i.e. the rest of the day’s) activities, ‘Classifier 1’ outputs were checked for both the left and right legs separately, to account for the possibility that a person could be moving one leg while still being considered to be in a ‘static’ posture. Data were not classified as dynamic unless the ‘Classifier 1’ output indicated that there was dynamic movement in both legs.

### Classifying postures (classifier 2)

For each sensor, the accelerometer data were further filtered by taking the moving mean over a 1s window. The filtered acceleration values were used to calculate the inclination angle of each sensor using Eq. (1), where $s_z$ is the moving mean of the acceleration in the z-axis and *g* is the magnitude of the moving mean of the acceleration when the participant is ‘standing still’.

The inclination angles of the three lower body sensors (Waist, Thigh and Ankle) were extracted and, along with their annotations, were fed into MATLAB’s ‘Classification Learner App’ to train a KNN classifier (Classifier 2). As mentioned earlier in "Static postures versus dynamic movements" section all the options in the ‘Classification Learner App’ were checked with the data and the classifier type which gave the highest accuracy was selected. The selected KNN classifier was a ‘Weighted’ KNN which uses a ‘Euclidean’ distance metric with 10 neighbours. To evaluate the model, 5-fold cross-validation and leave-one-subject-out cross validation were used.

## Results and discussion

### Classification of static and dynamic activities

The confusion matrices were examined for different combinations of features ((i) moving standard deviation of the magnitude of the thigh data alone, (ii) moving standard deviation of the magnitude of the thigh data combined with the moving standard deviation of the vertical axis of the thigh data, and (iii) the previous two features combined with the moving standard deviation of the magnitude of the waist data) and window sizes (0.5 s, 1 s, 1.5 s, 2 s and 3 s). They showed that the false positive and false negative values were gradually decreased with additional features when the windows were 0.5 s and 1 s. However, when the windows were 1.5 s, 2 s and 3 s, the classification accuracies for all combinations of features were 100% with the given training dataset.

Further, it was noted that the moving standard deviation of the magnitude of the waist data and of the thigh data were strongly correlated. As the thigh can capture more information than the waist in postural changes, only the standard deviation of the magnitude of thigh data was selected to train Classifier 1.

It was noted that for the static postures, the moving standard deviation did not surpass $\approx 0.005$ g, whereas for dynamic activities it was consistently above 0.1 g.

**Figure 3:**
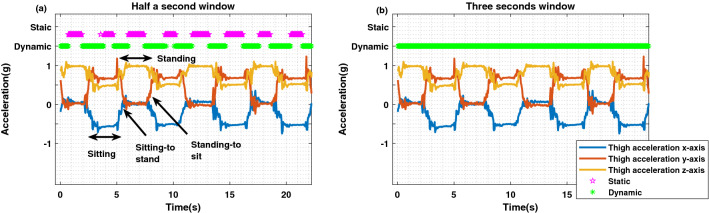
Classification outputs for a 0.5 s window (left) compared with a 3 s window (right), trained using the standard deviation of the magnitude of the thigh data. The plots show the 3-axes of acceleration from a sensor on the thigh as one participant performs 4 sit-to-stand cycles. The classifier outputs are shown at the top of each plot. For a 0.5 s window, the classifier identifies periods of static postures and dynamic movements within each sit-to-stand movement. In contrast, for a 3 s window, the whole segment is classified as a dynamic movement.

Figure 3 illustrates how the classification output varies with window size, over four sit-to-stand cycles. With window sizes of 0.5 s (left), 1 s, 1.5 s and 2 s, the classifiers identified periods of sitting and standing as static postures, and transitions between sitting and standing as dynamic movements. With a window size of 3 s (right), however, the classifier identified the whole segment where the participant was performing sit-to stands as a dynamic movement segment.

For this paper, the intention was to analyse the participants’ postural changes throughout the day and estimate the proportion of active (dynamic) movements relative to passive postures. Hence, the transition movements were not of primary interest, rather the focus was on identifying longer segments of static and dynamic activities. Therefore, the standard deviation of the magnitude of the ‘Thigh’ data for 3 s windows was selected as the only feature to train the classifier, based on the accuracy values as mentioned in section ‘Classification of static and dynamic activities’.

Classifier 1 was trained and evaluated with all 15 datasets across the 5 participants with 5-fold cross validation and the accuracy was 100%. Classifier 1 was further evaluated with a leave-one-subject-out approach. For each left-out participant, 3 days of data were used for testing, and roughly 90 seconds of data were taken from 4 static postures ($4~\text {postures} \times ~90~\text {s}\times 50~\text {Hz} \times 3 ~ \text {days} = ~54000$) and 2 dynamic activities ($2 ~\text {activities} \times ~90~\text {s}\times 50~\text {Hz} \times 3 ~ \text {days}~= ~27000$) to evaluate the classifier.

### Classification of postures

**Figure 4:**
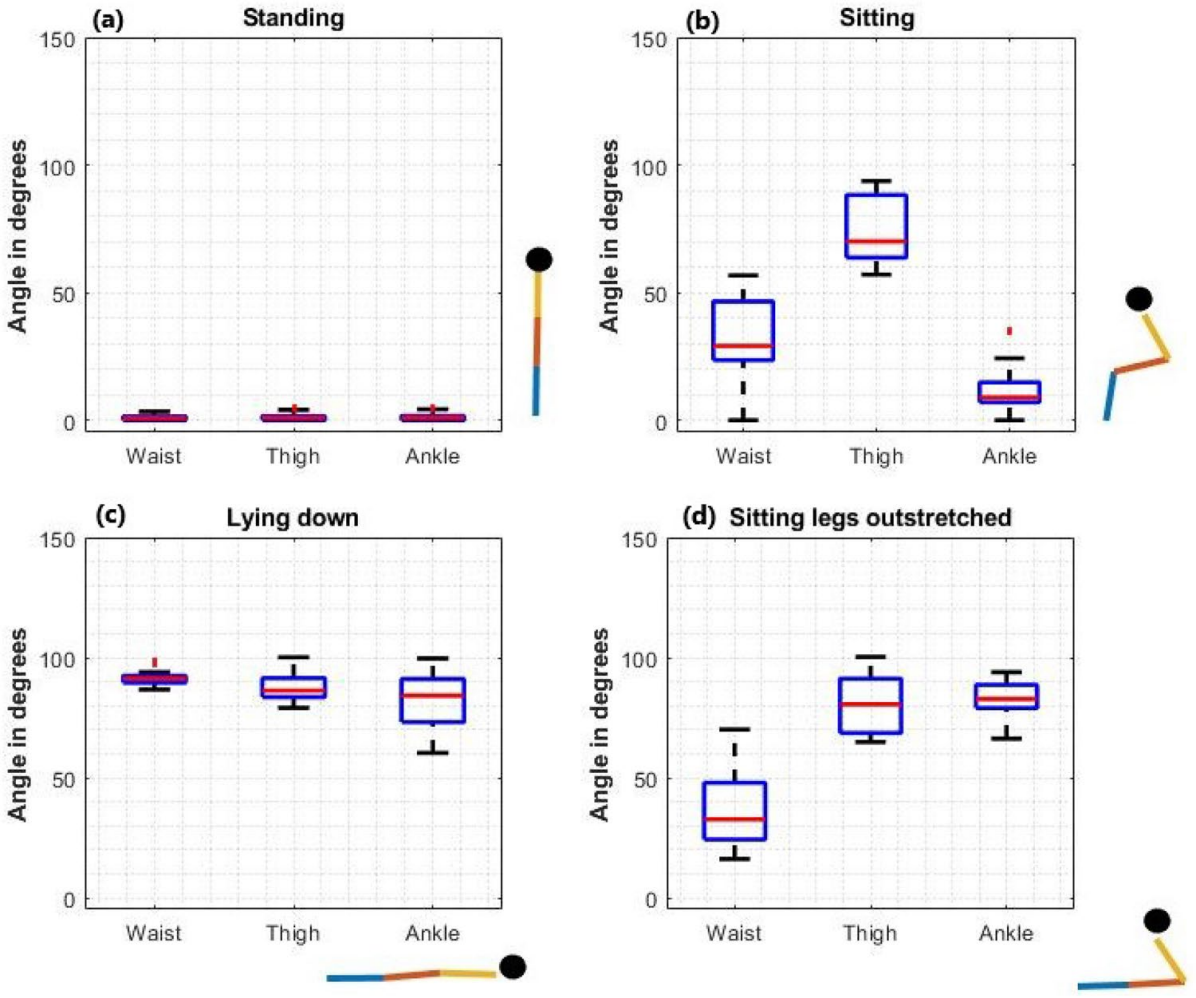
Inclination angles for the waist, thigh, and ankle from all 15 datasets for (**a**) standing, (**b**) sitting, (**c**) lying down, and (**d**) sitting with legs outstretched. The stick figures are drawn using the median value of the inclination angles.

**Figure 5:**
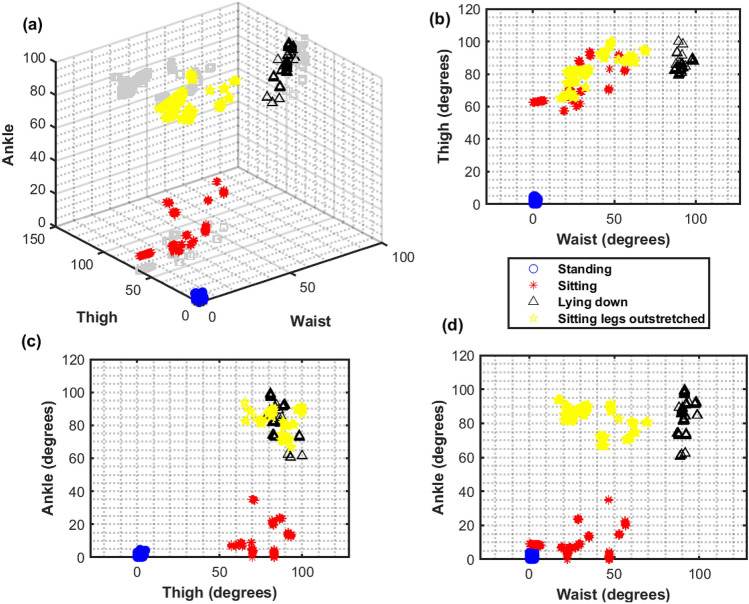
(**a**) 3D plot of the inclination angles. Shadows of the data are projected in grey onto the walls of the graph. Four clusters correspond to standing (blue ‘o’), sitting (red ‘x’), lying down (black ‘$\triangle$’) and sitting with legs outstretched (yellow pentagon). Projections of the data are shown in (**b**) ‘thigh’ vs ‘waist’, (**c**) ‘ankle’ vs ‘thigh’ and (**d**) ‘ankle’ vs ‘waist’.

The inclination angles for the waist, thigh, and ankle for each posture from all 15 datasets are shown in Fig. 4. Figure 5 shows the same data in a 3D representation, with the waist, thigh, and ankle inclination angles on the three axes. The plots show four clusters corresponding to the four postures.

Classifier 2 had a 100% classification accuracy for both 5-fold cross validation and leave-one-subject-out methods. The given confusion matrix in Table 1 was based on a 5-fold cross validation method.

**Table 1:** Confusion matrix for Classifier 2 (posture classification) Posture 1: Standing, Posture 2: Sitting,Posture 3: Lying down, Posture 4: Legs outstretched. There were approximately 22.5 minutes (67,500 samples) of data per activity (maximum of 90 seconds per posture per day x 15 participant-days).

True class	Posture 1	65785	0	0	0
	Posture 2	0	69015	0	0
	Posture 3	0	0	67175	0
	Posture 4	0	0	0	58605
		Posture 1	Posture 2	Posture 3	Posture 4
		Predicted class			

All the analyses presented above were conducted with the ‘Right’ leg data. The same analyses were conducted with the left leg to examine if there were any differences. Similar accuracies were observed from both Classifiers 1 and 2.

### Analysis of “usual activities”

By combining both classifier outputs the data were categorised into five categories, the four static postures plus dynamic movements as a fifth category. Again we observed that the accuracy remained the same at 100% even after combining both classifiers with the given dataset.

**Figure 6:**
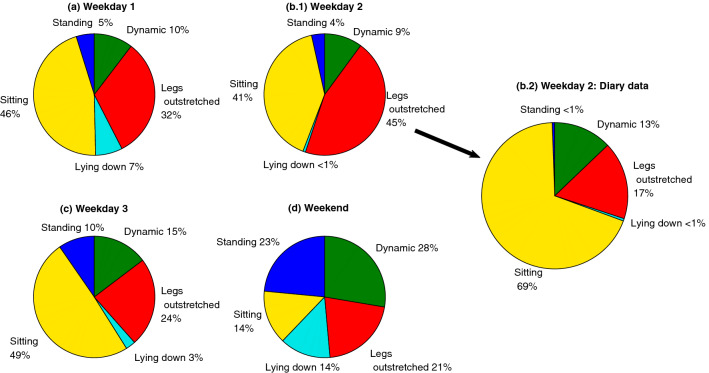
Four days’ activity summary report for ‘Participant A’, based on analysis of the sensor data (**a**, **b.1**, **c** and **d**) and activity percentages based on diary data for Weekday 2 (**b.2**). This includes three weekdays and one weekend day. Compared to weekdays, there was more standing and dynamic movements at the weekend. Also, the participant was sitting most of time during the weekdays with less time spent in dynamic movements.

The data collected from the participants’ “usual activities” for the rest of the day (i.e. non-ground truthed activities) were analysed to characterise the postural variations of the participants. Figure 6 shows one of the summary reports (‘Participant A’). Each day, ‘Participant A’ was wearing the sensors for more than 8 hours during daytime hours. Three of the days were weekdays when ‘Participant A’ was mainly working (in front of a computer) and during the weekend-day, the participant was doing miscellaneous activities including shopping, according to the diary reports.

By comparing the weekday plots against the weekend plot (Fig. 6a,b.1,c and d), the sensor data capture that ‘Participant A’ had been sitting and sitting with legs outstretched between 73% and 86% of the time throughout the data collection on weekdays. In contrast, on the weekend, the proportion of time spent sitting and sitting with legs outstretched was comparatively lower (35%). Moreover, the sensors captured a higher proportion of time spent lying down (14%) on the weekend, which corresponded to the participant having a nap and lying down on a sofa, according to their diary data. Further, during the weekend, the participant’s dynamic activities and standing durations made up a higher proportion of the activities (51%), compared to weekdays. During the weekdays the total dynamic and standing data were between 13% and 25%.

According to Fig. 6b.1, on Day 2, ‘Participant A’ had spent 45% of their day in a ‘sitting with legs outstretched’ posture and 41% of the time sitting. In order to check why ‘Participant A’ had been sitting with legs outstretched for a longer period than that of sitting, the data were analysed against the diary data. Figure 6b.2 shows the distribution of postures based on the classifier (b.1) as compared with the participant’s diary data (b.2). According to Fig. 6, it would appear that 28% of activity recorded in the diary as ‘sitting’ was classified as ‘sitting with legs-outstretched’, and roughly 5% of dynamic movements from the diary data appear to be classified as ‘standing’ data.

**Figure 7:**
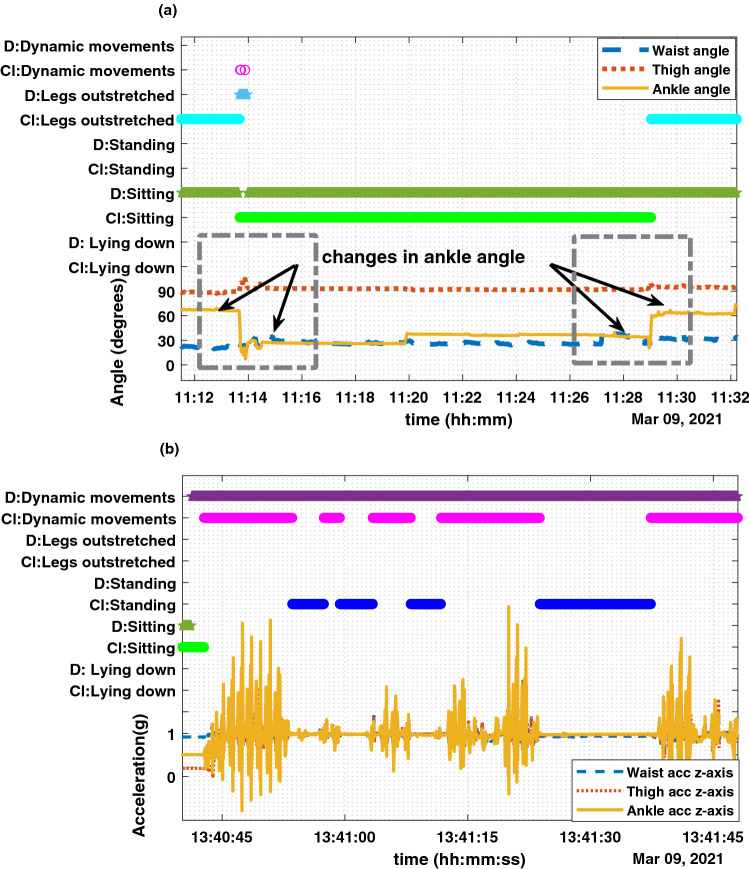
Two segments from Participant A’s Day 2 data with classifier output and diary data. Plot (**a**) shows the sensor to vertical angles of the waist (blue dash), thigh (red dot) and ankle (solid yellow) for a period of ‘sitting’ as per the diary record. The classifier output (Cl) and diary data (D) are shown in the upper part of the graph. The participant has only changed the ankle angle while the waist and thigh remain the same, resulting in the classifier output changing between ‘sitting’ and ‘sitting legs outstretched’. Plot (**b**) shows a period of fidgeting as per the diary data. The z-axis of the accelerometers on the waist (blue dash), thigh (red dot) and ankle (solid yellow) are shown. The classifier classified some segments as standing (shown in blue), while the diary recorded the whole segment as fidgeting (dynamic).

In order to understand these discrepancies better, the classifier output and the diary data were plotted alongside the corresponding angle data of the waist, thigh and ankle and the z-axis of acceleration from each sensor (Fig. 7).

Figure 7a shows a data segment where the classifier alternates between ‘sitting’ and ‘sitting with legs outstretched’ even though, that entire segment was recorded as ‘sitting’ in the diary. Within that segment, the ankle angles changed between $30^{\circ }$ and $60^{\circ }$, resulting in the classifier distinguishing them as ‘sitting’ and ‘sitting with legs outstretched’. When ‘Participant A’ was asked about the data, they stated that during some segments their legs were in different positions and they might have stretched their legs while working. By considering all these factors it could be said that unconsciously the participant might have stretched out the ankles from the proper sitting posture without changing the waist or thigh, which could be the reason for the discrepancies in ‘sitting ’ vs ‘sitting with legs outstretched’ data between the classifier output and the diary data.

Similarly, Fig. 7b shows an instance where there was a discrepancy in standing and dynamic activities in classifier output versus diary data. The participant recorded a period of fidgeting (from 13:40) which was annotated as dynamic activity. Examination of the sensor data, however, indicated that the data also included periods of standing which were not noted in the diary, yet were classified by the classifier as standing. Figure 7b shows one such fidgeting segment from the whole dataset. Not only by considering the classifier output, but also by examining the flat lines of the waist, thigh and ankle accelerometer signals, it can be said that there were static (standing) segments within that fidgeting segment.

This paper focused on analysis of static postures based on accelerometry. Further, this paper gives an insight as to how the different window sizes can be used in feature vectors in training classifiers under different scenarios. Both classifiers implemented in this study had 100% accuracy when trained and evaluated with an annotated dataset. This dataset was highly-selected and accuracies could decline with more naturalistic activities that include, for example, sitting with the legs crossed. Nevertheless, the results indicate that sensors embedded in loose clothing are significant as a way of capturing posture information. Comparison of the diary data and the posture classifier output indicates that a significant percentage of activity during a person’s days can be captured by postures that can be recognised with sensors only on the lower body (waist, thigh, ankle). Still, inclusion of upper body sensor data could be useful to understand whole body postures.

Even though this study is limited to the classification of data into four basic postures, further improvements are possible by using a different method for estimating the inclination angle. For instance, a rotation matrix approach as described in "Estimating sensor inclination angle with a rotation matrix" section could be used, which would allow estimation of angles from $0^{\circ }$ to $360^{\circ }$ (versus the arccos method which estimates angles between $0^{\circ }$ and $180 ^{\circ }$). With some adjustments to the method of estimating the inclination angles to be able to distinguish, for example, between “forward” and “backward” inclinations, there is a possibility of using these clothing-mounted wearable sensors in analysing ‘sleeping’ postures such as “supine position”, “prone position”, “right position”, “left position”, and “sitting position” as mentioned in^30^.

We acknowledge that this study was conducted with a limited number of participants, nevertheless the data collection with each of the 5 participants was extensive (1–4 full days each, resulting in more than 90 hours of data) and systematic. Altogether, 15 datasets (i.e. 15 participant-days) were used to train and evaluate the classifiers, and we believe that this analysis is sufficiently robust to show that the clothing-mounted sensor data can be used productively in posture analysis.

Dynamic activity classification is as important as posture classification, and is an important direction for future work. One of the main benefits of looking at dynamic activities is the possibility of analysing the intensity of physical activities. Physical inactivity causes many health issues^31^ and classification of dynamic activities could help provide insights that are relevant in healthcare monitoring. Another area for further work is to extend this study to analyse the upper body data, to improve the static posture and dynamic movement classifications.

## Conclusion

Monitoring posture and classifying activities for long-term healthcare can be challenging. In order to achieve a reasonable level of accuracy, more sensors can help but can be difficult or cumbersome for the person to wear. A solution is to mount the sensors into everyday clothing, so the data collection is unobtrusive for the individual. This study analysed data from 3 sensors mounted along the lateral seam of both legs of loose-fitting trousers, corresponding to the waist, thigh, and ankle. Three features (inclination angles of the waist, thigh, and ankle) were used to implement a posture classifier, which achieved 100% accuracy. Hence, we conclude that sensors mounted on loose clothing can be used successfully for posture classification.

## Supplementary Information

Supplementary Information 1.

Supplementary Information 2.

## Data Availability

All data analysed in this paper are included in this article (and its supplementary information files).

## References

[1] Cochrane W (1924). The importance of physique and correct posture in relation to the art of medicine. Br. Med. J..

[2] Wong F, Liu P, Allidina Y, Blendis L (1996). The effect of posture on central blood volume in patients with preascitic cirrhosis on a sodium-restricted diet. Hepatology.

[3] Skelton DA, McLaughlin AW (1996). Training functional ability in old age. Physiotherapy.

[4] Bonomi AG, Goris A, Yin B, Westerterp KR (2009). Detection of type, duration, and intensity of physical activity using an accelerometer. Med. Sci. Sports Exerc..

[5] Baptista, R., Antunes, M., Aouada, D., Ottersten, B. *et al.* Flexible feedback system for posture monitoring and correction. In *2017 Fourth International Conference on Image Information Processing (ICIIP)*, 1–6 (IEEE, 2017).

[6] Benaim C, Pérennou DA, Villy J, Rousseaux M, Pelissier JY (1999). Validation of a standardized assessment of postural control in stroke patients: The postural assessment scale for stroke patients (pass). Stroke.

[7] Estrada-Barranco C, Cano-de-la Cuerda R, Abuín-Porras V, Molina-Rueda F (2021). Postural assessment scale for stroke patients in acute, subacute and chronic stage: A construct validity study. Diagnostics.

[8] Mosenia A, Sur-Kolay S, Raghunathan A, Jha NK (2017). Wearable medical sensor-based system design: A survey. IEEE Trans. Multi-Scale Comput. Syst..

[9] Attal F (2015). Physical human activity recognition using wearable sensors. Sensors.

[10] Maurer, U., Smailagic, A., Siewiorek, D. P. & Deisher, M. Activity recognition and monitoring using multiple sensors on different body positions. In *International Workshop on Wearable and Implantable Body Sensor Networks (BSN’06)*, 4–pp (IEEE, 2006).

[11] Lugade V, Fortune E, Morrow M, Kaufman K (2014). Validity of using tri-axial accelerometers to measure human movement-part i: Posture and movement detection. Med. Eng. Phys..

[12] Lyons G, Culhane K, Hilton D, Grace P, Lyons D (2005). A description of an accelerometer-based mobility monitoring technique. Med. Eng. Phys..

[13] Kang S-W (2017). The development of an imu integrated clothes for postural monitoring using conductive yarn and interconnecting technology. Sensors.

[14] Mokhlespour Esfahani MI, Nussbaum MA (2019). Classifying diverse physical activities using “smart garments”. Sensors.

[15] Skach S, Stewart R, Healey PG (2019). Smarty pants: Exploring textile pressure sensors in trousers for posture and behaviour classification. Multidiscipl. Digi. Publ. Inst. Proc..

[16] Van Laerhoven, K., Schmidt, A. & Gellersen, H.-W. Multi-sensor context aware clothing. In *Proceedings. Sixth International Symposium on Wearable Computers,*, 49–56 (IEEE, 2002).

[17] Harms, H., Amft, O. & Tröster, G. Modeling and simulation of sensor orientation errors in garments. In *Proceedings of the Fourth International Conference on Body Area Networks*, 1–8 (2009).

[18] Chiuchisan, I., Geman, O. & Hagan, M. Wearable sensors in intelligent clothing for human activity monitoring. In *2019 International Conference on Sensing and Instrumentation in IoT Era (ISSI)*, 1–4 (IEEE, 2019).

[19] Lin, Q. *et al.* E-jacket: Posture detection with loose-fitting garment using a novel strain sensor. In *2020 19th ACM/IEEE International Conference on Information Processing in Sensor Networks (IPSN)*, 49–60 (IEEE, 2020).

[20] Fida B, Bernabucci I, Bibbo D, Conforto S, Schmid M (2015). Varying behavior of different window sizes on the classification of static and dynamic physical activities from a single accelerometer. Med. Eng. Phys..

[21] Chong J, Tjurin P, Niemelä M, Jämsä T, Farrahi V (2021). Machine-learning models for activity class prediction: A comparative study of feature selection and classification algorithms. Gait Posture.

[22] Jayasinghe U, Harwin WS, Hwang F (2019). Comparing clothing-mounted sensors with wearable sensors for movement analysis and activity classification. Sensors.

[23] Weisstein, E. W. Rotation formula. https://mathworld.wolfram.com/ (2009).

[24] Filippeschi A (2017). Survey of motion tracking methods based on inertial sensors: A focus on upper limb human motion. Sensors.

[25] Craig JJ (2005). Introduction to Robotics: Mechanics and Control.

[26] Mahony R, Hamel T, Pflimlin J-M (2008). Nonlinear complementary filters on the special orthogonal group. IEEE Trans. Autom. Control.

[27] Madgwick, S. O., Harrison, A. J. & Vaidyanathan, R. Estimation of imu and marg orientation using a gradient descent algorithm. In *2011 IEEE international conference on rehabilitation robotics*, 1–7 (IEEE, 2011). 10.1109/ICORR.2011.5975346 22275550

[28] Sabatini AM (2011). Estimating three-dimensional orientation of human body parts by inertial/magnetic sensing. Sensors.

[29] Brugman, H., Russel, A. & Nijmegen, X. Annotating multi-media/multi-modal resources with ELAN. In *LREC*, 2065–2068 (2004).

[30] Kishimoto, Y., Akahori, A. & Oguri, K. Estimation of sleeping posture for m-health by a wearable tri-axis accelerometer. In *2006 3rd IEEE/EMBS International Summer School on Medical Devices and Biosensors*, 45–48 (IEEE, 2006).

[31] Parkka, J. *et al.* Estimating intensity of physical activity: A comparison of wearable accelerometer and gyro sensors and 3 sensor locations. In *2007 29th annual international conference of the IEEE engineering in medicine and biology society*, 1511–1514 (IEEE, 2007). 10.1109/IEMBS.2007.4352588 18002254

