# Supplementary material for: Classification of static postures with wearable sensors mounted on loose clothing
Source: Sci Rep. 2023 Jan 4;13:131. doi: 10.1038/s41598-022-27306-4 (PMC9812968; doi:10.1038/s41598-022-27306-4)
Supplement: Supplementary file 1 — Supplementary Information 1. [file 41598_2022_27306_MOESM1_ESM.pdf]

Paper title: Classification of Static Postures with Wearable Sensors Mounted on Loose Clothing (Scientific Reports))

Authors: Udeni Jayasinghe[1,2,*], Balazs Janko[3], Faustina Hwang[1,†], and William Harwin[1,†]

Study	Sensor type	Sensor placement			Sensors mounted on		Static vs dynamic classification		Posture classification	
		**Waist**	**Thigh**	**Ankle**	**Body**	**Clothing**	**No of features**	**Classification method**	**No of features**	**Classification method**
Lyons et al. [12]	2 Dual axis accelerometers	√	√	-	√	-	1	thresholding	2 inclination angles	thresholding
Vipul et al. [11]	2 Triaxial accelerometers	√	√	-	√	-	1	thresholding	2 inclination angles	thresholding
Fida et al. [20]	1 Triaxial accelerometer	√	-	-	√	-	22	Machine learning	-	
Chiuchisan et al. [18]	2 Inductive sensors on an Arduino Nano Board	-	knee	-	-	√ Loose trousers similar to Hospital staff trousers	1	thresholding	-	
Skach et al. [15]	Textile pressure sensors		Thigh and buttocks area		-	√ Smart garment	-		1	Machine learning – Random forest
Lin et al. [19]	4 strain sensors	√	shoulder, elbow, abdomen			√ Loose fitting E-jacket	1	Long Short-Term Memory (LSTM) networks	1	Long Short-Term Memory (LSTM) networks
Present study	3 accelerometers	√	√	√	-	√ Every day loose clothing	1	Machine learning (KNN)	3 inclination angles	Machine learning – (KNN)

Table: Comparison of prior studies with the present study.
